# The involvement of T cell pathogenesis in thyroid-associated ophthalmopathy

**DOI:** 10.1038/s41433-018-0279-9

**Published:** 2018-12-07

**Authors:** Yazhuo Huang, Sijie Fang, Dan Li, Huifang Zhou, Bin Li, Xianqun Fan

**Affiliations:** 1 Department of Ophthalmology, Shanghai Ninth People’s Hospital, Shanghai JiaoTong University School of Medicine, 200011 Shanghai, China; 2 Shanghai Key Laboratory of Orbital Diseases and Ocular Oncology, 200011 Shanghai, China; 3 Shanghai Institute of Immunology, Shanghai JiaoTong University School of Medicine, 200025 Shanghai, China; 4 Department of Immunology and Microbiology, Shanghai JiaoTong University School of Medicine, 200025 Shanghai, China

## Abstract

Thyroid-associated ophthalmoapthy (TAO) is the most common orbital disease. As an autoimmune disorder, it is caused by self-reactive lymphocytes that escape immune tolerance, but the mechanism is not fully understood. The basic process of TAO is the infiltration of immune cells in orbital tissues, the activation of orbital fibroblasts (OFs), and the proliferation and differentiation of OFs and lymphocytes. Activated OFs secrete inflammatory regulators, growth factors, and chemokines, thereby maintaining and amplifying the immune responses. The interactions between OFs and lymphocytes lead to the expansion and the remodeling of the orbital tissues, presenting the clinical manifestations of TAO. This review will focus on the role of T cell subsets (Type 1, Type 2, Type 17 helper T cells, and regulatory T cells) in the pathogenesis of TAO. However, we still need further studies to unravel the pathogenesis, to confirm current hypotheses, and to provide novel ideas for appropriate clinical treatment of TAO.

## Introduction

Graves’ orbitopathy (GO), named after its being the most common extrathyroidal complication of Graves’ disease (GD), also known as thyroid-associated ophthalmopathy (TAO) [1, 2], is an autoimmune disorder, which is found in 25–50% patients with GD, 2% patients with chronic thyroiditis, and some euthyroid cases [3]. Its main manifestations are eyelid retraction, diplopia (caused by extraocular muscle dysfunction), protrusion, periorbital edema, conjunctival hyperemia, exposure keratitis, and compressive optic neuropathy [4, 5]. The physical discomfort caused by craniofacial deformity and visual impairment in TAO has a continuous negative impact on patients’ quality of life [6].

Previous studies have shown that TAO is an organ-specific disease, which is affected by multiple factors including genetics, environment, and smoking [3, 7]. Meanwhile, the hypothesis that the T cell-mediated immunity contributes to TAO development has been widely accepted [8]. In order to gain a deeper understanding of the immune mechanism responsible for TAO, it is necessary to analyze the function of different T cells and their cytokine profiles. This review mainly focuses on the role of CD4^+^ T cell subtypes (Type 1, Type 2, Type 17 helper T cells, and regulatory T cells) in the pathophysiology of TAO based on previous and recent studies. The elucidation of T cell immunity in TAO may provide thought-provoking ideas for developing effective treatment.

## T cells

### Brief introduction

T cells are developed and differentiated from bone marrow-derived lymphoid stem cells in the thymus, occupying 65–75% of peripheral blood lymphocytes [9]. According to the type of T cell receptor, T cells can be divided into αβT cells and γδT cells. The former ones account for the vast majority of T cell population. In the thymus, T cells undergo positive and negative selection and differentiate into either CD4^+^ T cells or CD8^+^ T cells. The CD4^+^ T cells are helper T cells (Th), playing a leading role in cellular immunity and contributing to humoral immunity. They can be used to assess the status of the immune system [10]. The CD8^+^ T cells are cytotoxic T cells (Tc/CTL) that are primarily responsible for immune defense against intracellular pathogens and tumor monitoring [11]. Under normal conditions, the stability and balance of CD4/CD8 ratio is an important factor for the body’s immune function [12], while the T cell subtypes remain at certain proportion.

### T cells in TAO

According to previous studies, T cells and their cytokines may participate in the pathogenesis of TAO through the following pathways: (1) Activate B cells and stimulate the production of autoantibodies. When autoimmune tolerance in TAO is disrupted, antigen-presenting cells that recognize the autoantigen thyroid-stimulating hormone receptor (TSHR) expressed on orbital fibroblasts (OFs) activate T cells. Meanwhile, B cells migrate to the orbit and recognize TSHR through B cell receptor, which is the first signal of B cell activation. The second signal of B cell activation is provided by activated T cells through the combination of CD40L on T cell surface and CD40 on B cell surface. This interplay also stimulates T cells to secrete cytokines such as interleukin (IL)-4, which is essential for further activation of B cells and antibody class switching [5, 13]. Activated B cells undergo clone proliferation and differentiate into plasma cells that produce autoantibodies. These autoantibodies (including stimulating, blocking, and neutralizing subtypes) recognize and attack adipose connective tissues in the orbit. (2) Promote the expression of adhesion molecules. The interaction of B7 on B cell surface with CD28 on T cell surface provides the second signal for T cell activation [5, 13]. Activated T cells, primarily CD4^+^ T cells, produce a variety of adhesion molecules. Together with the chemokines and adhesion molecules secreted by stimulated OFs, these factors mediate the recruitment of more lymphocytes into orbital tissues and the further interaction between OFs and T cells [14, 15]. (3) Produce inflammatory cytokines. Cytokines produced by CD4^+^ T cells aggravate the immune responses of TAO by amplifying and maintaining orbital inflammation. They also promote the proliferation and differentiation of OFs, ultimately leading to glycosaminoglycan deposition, orbital fibrosis, and adipose hyperplasia, namely orbital tissue remodeling.

### Effect of cytokines and chemokines on TAO

Fibroblasts located in the connective tissues of orbit are called OFs and have been identified as target cells in TAO [16–19].

OFs play a vital role in lymphocyte infiltration and B cell differentiation. Many studies have revealed the regulation of OFs by various cytokines and growth factors in TAO. IFN-γ stimulates OFs to secret monocyte chemotactic factors such as C–C motif ligand (CCL) 2 and T cell chemokines, including C–X–C motif ligands (CXCL) 9, CXCL10, and CXCL11 [20, 21]. In TAO, once the insulin-like growth factor receptor-1 pathway is activated by IL-1β and IgGs, OFs can produce IL-16 and regulated on activation, normal T cell expressed and secreted (RANTES) [4]. IL-1β also up-regulates the expression of cyclooxygenase-2 by enhancing its gene promoter activity and mRNA stability in OFs [68]. The up-regulation of cyclooxygenase-2 promotes the synthesis of prostaglandin E_2_ (PGE_2_) [70]. Leukoregulin also stimulates the production of PGE_2_ in OFs [69].

Binding of PGE_2_ to the PGE_2_ receptor expressed on OFs stimulates the expression of a large amount of cAMP by OFs, thus resulting in the secreation of IL-6 [71]. In TAO, PGE_2_ promotes the maturation of B cells and facilitates antibody class switching. It also affects the differentiation of T cells, activates the degranulation of mast cells, and induces a Th2-type immune response [4, 15].

CD40L promotes the synthesis and secretion of hyaluronic acid, IL-6, IL-8, and CCL2 in OFs [13, 14, 22]. IL-6 can facilitate the synthesis of immunoglobulins, the development of plasma cells, the production of IL-4 and the differentiation of T cell subsets into Th2 cells. CCL2 and IL-8 are potent monocyte chemotactic factors that enhance the infiltration of monocytes into orbital connective tissues of TAO patients. Other cytokines such as TNF-α and growth factors such as platelet-derived growth factor (PDGF)-AA, PDGF-AB, and PDGF-AC also stimulate OFs to express CCL2, CCL5, CCL7, IL-6, IL-8 and IL-16,which are involved in the recruitment and activation of T cells, B cells, and mast cells [15].

In orbital tissues, the infiltration and activation of lymphocytes depend not only on the concentration of chemokines in orbital microenvironment, but also on adhesion molecules and costimulatory molecules expressed on lymphocytes, endothelial cells, and histocytes. The expression of intercellular adhesion molecule (ICAM)-1 on OFs can be increased by IL-1α, IL-1β, IFN-γ, and TNF-α. Early studies have found that the level of CD40 in OFs of patients with TAO are higher than in healthy controls, and can be further elevated by IFN-γ stimulation [14]. In TAO, the combination of CD40 and CD40L not only enhances the secreation of CCL2, IL-6, IL-8, IL-1α, and PGE_2_, but also promotes the expression of ICAM-1 [15].

## CD4^+^ T cells

The CD4^+^ T cells can be divided into subtypes including T helper (Th)1, Th2, Th17, T follicular helper (Tfh), regulatory T (Treg), Th9, and Th22 cells. Th1 cells mainly mediate cellular immunity and delayed hypersensitivity inflammation through the production of inflammatory cytokines. Th2 cells drive B cells to produce antibodies in humoral immune responses. Th17 cells, named for their secretion of IL-17, are resistant to pathogenic microbial infections and play a role in human autoimmune disease attack [23, 24]. Treg cells are T cell subsets with immunoregulatory functions [24, 25]. Tfh cells are named after their location in lymphoid follicles. They promote B cell differentiation and memory cell production [26]. Th9 cells, mainly located in peripheral blood and skin tissues of patients with hypersensitivity diseases, secret IL-9 [27]. Th22 cells produce IL-22 and infiltrate the epidermis of patients with inflammatory dermatosis [28].

### Th1 and Th2 cells

Previous studies have shown that at least two helper T cell groups are involved in the development of TAO: Th1 cells and Th2 cells. The relationship between the imbalanced Th1/Th2 ratio and human autoimmune diseases has been a hot topic of research [29–31]. Many studies have focused on Th1/Th2 subsets and their its related cytokines in the pathogenesis of TAO. However, some findings are contradictory.

Xia et al. [8] compared the difference of Th1/Th2 balance in the peripheral blood of TAO patients and GD patients, and found that the proportion of Th1 cytokines in TAO patients was significantly higher than that in GD patients, In addition, the frequency of Th1 cells and the Th1/Th2 ratio were positively correlated with the inflammatory activity score of TAO. Therefore, Th1 type cytokines are thought to play a Key role in inducing the immunopathological mechanism of TAO. Pappa et al. [32] studied 17 extraocular muscle specimens of TAO patients and found that most of the infiltrating T cells were CD4^+^ and both Th1 and Th2 cytokines were detected. Antonelli et al. [33] analyzed cytokine secretion in orbital myocytes and adipocytes cultured from TAO patients in vitro and found that Th1 type cytokines dominated both cells. Hiromatsu et al. [34] examined cytokine gene expression in muscles and adipose tissues of TAO patients, and found that the orbital muscle tissue were dominated by Th1 cytokines, while cytokine types in orbital fat tissues varied from person to person. In these studies, *ifng* expression was observed in the extraocular muscles of all TAO patients, indicating that Th1 type cytokines play an important role in the initiation of TAO development.

Wakelkamp et al. [35] cultured OFs from both active and inactive TAO patients. They found that active TAO patients was characterized by Th1 type cytokines and there was no direct correlation between Th2 type cytokines and disease progression. Han et al. [36] further demonstrated that IFN-γ (Th1 type cytokines) and IL-4 (Th2 type cytokines) had the same effect on the production of hyaluronic acid and PGE_2_ by OFs cultured in vitro. They also observed that the orbital tissues of TAO patients with hyperthyroidism for less than 2 years were mainly infiltrated by Th1 cells, while those with TAO for more than 2 years were predominately infiltrated by Th2 cells. It can be speculated that the early stage of TAO is mainly caused by cell-mediated immune responses, while the late stage of TAO is a humoral-mediated immune response, that is, Th1 cells play a significant role in inducing the pathological process of TAO, and the chronic phase of TAO is dominated by Th2-type immune responses.

### Th17 cells

Th17 cells is a newly identified CD4^+^ T cell subset in recent years, which are related to various autoimmune diseases, including rheumatoid arthritis, multiple sclerosis, psoriasis, inflammatory bowel disease, Behcet’s disease, systemic sclerosis, systemic lupus erythematosus, and high IgE syndrome [37]. These autoimmune diseases are regulated by the IL-23/IL-17A axis. IL-23, secreted by dendritic cells and macrophages, maintains the phenotype and function of Th17 cells [38]. Retinoic acid receptor-related orphan receptor-γt and signal transducer and activator of transcription 3 are key regulators of the transcription of *il17a* and the differentiation of Th17 cells. Mature Th17 cells, constitutively expressing IL-23R, mainly produce IL-17A, IL-17F, and IL-22. IL-17A has been identified as an important pro-inflammatory cytokine. Moreover, IL-17A can also be produced by CD8^+^ T cells, γδT cells, and natural killer cells [38–40].

In GD, the frequency of Th17 cells and the level of IL-17A in the peripheral blood of patients were significantly increased [41], while the changes of single nucleotide polymorphism of *il17a* are related to GD susceptibility [42, 43]. In TAO patients, the level of IL-17A and the number of IL-17A-producing T cells in the peripheral blood were higher than those of healthy controls [44–47]. Furthermore, a large amount of IL-17A was detected in the tears of TAO patients [48, 49]. Taken together, these results indicated that the CD4^+^ Th17 cells may contribute to the immunopathological process of TAO. IL-17A was also shown to promote inflammation and fibrosis of OFs derived from TAO patients [47] and to promote the expression of regulated on activation, normal T cell expressed and secreted (RANTES) by OFs with the assistance of CD40L [50]. The study from our group demonstrated the possible interplay between Th17 cells and OFs: Th17 cells stimulated the expression of proinflammatory cytokines (IL-6, IL-8, MCP-1, TNF-α, and GM-CSF) and costimulatory molecules (CD40 and MHC II) of OFs and regulated the fibrosis and adipogenesis of OFs subsets; at the same time, OFs triggered Th17 cell differentiation and function via PGE_2_ secreion[51]. Recent studies showed that CD34^+^ fibrocytes may penetrate into orbital tissues, differentiate into CD34^+^ OFs under inflammatory conditions, and participate in TAO autoimmunity and tissue remodeling [5, 52]. We further found that in TAO autologous C-C chemokine receptor type 6^+^ Th17 cells facilitated the inflammatory and antigen-presenting functions of those CD34^+^ fibrocytes and fibrocytes in turn recruited Th17 cells in a macrophage inflammatory protein 3/C-C chemokine receptor type 6-dependent manner [53].

In autoimmune diseases, γδ T cells have immunoregulatory properties and also secrete IL-17A [54]. A previous report showed that orbital infiltrating T cells were primarily γδT cells, while the αβT helper cells were rare [55]. Intriguingly, a confirmed subset of IL-17A-producing γδT cells was detected in the circulation of TAO patients [47]. Further studies are needed to explore which T cell subset contributes to the elevated IL-17A levels in the circulation, tears, and orbital tissues of TAO patients.

### Treg cells

Treg cells account for 5–10% of peripheral blood CD4^+^T cells. They are described as CD4^+^CD25^+^ T cells, a subset with immunoregulatory functions, playing a role in controlling autoimmunity, tumor immunity, and transplantation tolerance. As a Key transcriptional factor of Treg cells, forkhead box P3 controls the development and function of Treg cells [56]. Currently, Treg cell dysfunction has been shown to be an important risk factor for the pathogenesis of various autoimmune diseases, which also contributes to the development of autoimmune thyroid disease [57–59]. However, the role of Treg cells in the development of TAO remains unclear. A previous study analyzed the peripheral blood of one TAO patient before and after treatment with rituximab and found an increase in the number of Treg cells after treatment, which might attribute to B cell depletion partially [60]. Another study showed that patients with improved TAO were more likely to have higher frequencies of Treg cells than those with stable or deteriorated TAOs. Thus the number of Treg cells in peripheral blood of TAO patients can be used as a predictior of clinical course [61]. Kahaly *et al*. demonstrated that the proportion of Treg cells in the peripheral blood leukocytes derived from TAO patients increased after incubation with rabbit polyclonal anti-T lymphocyte globulin [67]. These results indicate that the number of Treg cells may be related to the severity of inflammatory responses in TAO.

Cytotoxic T lymphocyte antigen 4 (CTLA-4), constitutively expressed on Treg cells, is an immunological checkpoint for negative regulation of T cell activation [62]. It has been previously reported that the mRNAs of CTLA-4 were lower in orbital tissues and peripheral blood of patients with severe TAO compared with patients with mild TAO, confirming the involvement of CTLA-4 in TAO autoimmunity[63, 64]. However, forkhead box P3 mRNA levels were higher in orbital tissue of TAO patients compared with those from healthy controls and were positively correlated with TAO severity. Altogether, the function abnormality of Treg cells is observed in TAO patients, which may be associated with the expansion of the orbital inflammation, especially in patients with severe TAO.

## CD8^+^ T cells

Similar to CD4^+^ T cells, the CD8^+^ T cells are heterogenous and can be divided into Tc1 secreting IFN-γ, Tc2 secreting IL-5, Tc9 secreting IL-9, Tc17 secreting IL-17, and CD8^+^ Treg secreting TGF-β. CD8^+^ T cells mediate and suppress cell-mediated immune responses [65]. It has been demonstrated that the CD8^+^ T cells participate in most autoimmune disorders and post-transplantation [66]. Reduction and dysfunction of CD8^+^ T cells lead to impaired immune surveillance and Th cells which respond to autoantigens will increase and the autoimmune responses be amplified. A previous study showed an increased ratio of CD4/CD8 in peripheral blood mononuclear cells of patients with GD [8] Another study demonstrated higher frequency of CD4^+^ and CD4/CD8 ratio together with decreased CD8^+^ T cells in early TAO and GD without orbitopathy [67]. An earlier study from Grubeck-Loebenstein *et al*. examined six T cell lines from the orbital tissues of TAO patients and found that they were CD8^+^ T with both of Th1 and Th2 type cytokines in situ [17]. Considering that patients involved in Grubeck– Loebenstein′s research had longstanding histories of GD, the conflict between these results may be due to glucocorticoid therapy or the self-limitation of the disease. However, little is known about the pathogenic mechanism of CD8^+^ T cells in TAO.

## Conclusion

**Fig. 1:**
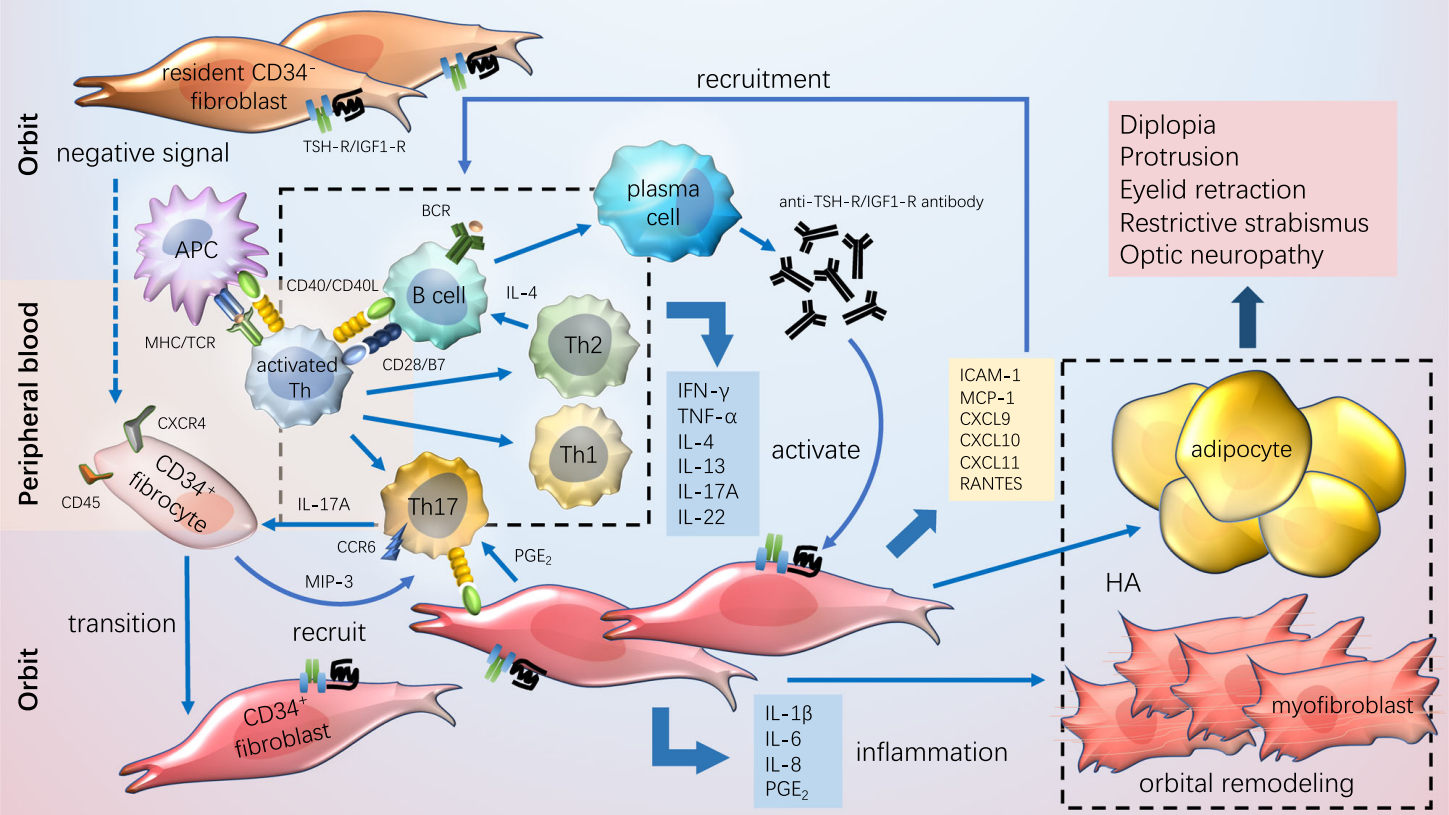
Pathogenesis of TAO. In TAO, T cells, B cells, and CD34^+^ fibrocytes infiltrate the orbit. Antigen- presenting cells present self-antigens to T cells and activate T cells. Activated T cells differentiate into subsets including T helper (Th) 1, Th2, and Th17 cells, producing cytokines like IFN-γ, TNF-α, IL(interleukin)-4, IL-13, IL-17A, and IL-22, which exacerbate immune inflammatory responses, activate OFs and stimulate proliferation and differentiation of orbital fibroblasts (OFs). The self-antigen is the first signal to activate B cells and the second signal is provided by activated T cells. Activated B cells differentiate into plasma cells that secrete autoantibodies. Fibrocytes can recruit Th17 cells in a macrophage inflammatory protein 3/C-C chemokine receptor type 6-dependent manner, and can also differentiate into CD34^+^ fibroblasts, which coexist with the resident CD34^−^ OFs in orbit. Both CD34^+^ and CD34^−^ OFs express the thyroid stimulating hormone receptor and insulin-like growth factor 1 receptor. Activated OFs that secrete chemokines including intercellular adhesion molecule-1, macrophage inflammatory protein 1, C-X-C motif ligand (CXCL) 9/10/11, and regulated on activation, normal T cell expressed and secreted (RANTES) can recruit more lymphocytes. They also secrete proinflammatory factors such as IL-1β, IL-6, IL-8, and prostaglandin E_2_, maintaining and amplifying immune responses. Activated OFs differentiate into adipocytes, myofibroblasts, and promote the synthesis of hyaluronic acid, all of which contribute to the increase in the volume of the orbital tissues and remodeling of the orbit, ultimately leading to the clinical manifestations of TAO.

There are considerable obstacles in studying the role of T cells in TAO. First, although peripheral blood of patients with TAO is readily available, the proportion and function of circulating T cells might not fully reflect the inflammatory responses within the orbit. Meanwhile, due to the difficulty in obtaining orbital connective tissue specimens of early or active TAO patients without immunomodulation therapy and the lack of stable TAO animal models, the analysis of T cells infiltrating the retrobulbar tissues is usually performed using the specimens from longstanding TAO patients or patients who have experienced multiple treatments. The T cell lines cultured from orbital tissues are limited and there exist certain biases after mitogen stimulation in vitro. Despite these difficulties, researchers have outlined the role of T cells in the pathogenesis of TAO in recent decades. A summary of current models for the pathogenesis of TAO is depicted in Figure 1, including the major processes of disease progression, such as costimulatory signals, adhesion factors, and cytokines that drive lymphocyte migration, activation, and immune responses to TSHR. Treatment strategies targeting these processes could provide new opportunities to improve the therapeutic effects and prevention of TAO.
